# Supplementary material for: FERN – a Java framework for stochastic simulation and evaluation of reaction networks
Source: BMC Bioinformatics. 2008 Aug 29;9:356. doi: 10.1186/1471-2105-9-356 (PMC2553347; doi:10.1186/1471-2105-9-356)
Supplement: Additional file 1 — FERN distribution, Version 1.3. This archive contains the FERN source code and binaries as well as documentation and example models in FernML and SBML. [file 1471-2105-9-356-S1.zip › fern/doc/javadoc/fern/simulation/algorithm/GibsonBruckSimulator.html]

GibsonBruckSimulator


---


|  |  |  |
| --- | --- | --- |
| **Overview** \| **Package** \| **Class** \| **Use** \| **Tree** \| **Deprecated** \| **Index** \| **Help** | |  |
| **PREV CLASS**   **NEXT CLASS** | **FRAMES**    **NO FRAMES**     **All Classes** |  |
| SUMMARY: NESTED \| FIELD \| CONSTR \| METHOD | DETAIL: FIELD \| CONSTR \| METHOD |  |


---


## fern.simulation.algorithm Class GibsonBruckSimulator

```
java.lang.Object
  fern.simulation.Simulator
      fern.simulation.algorithm.GibsonBruckSimulator
```

---

``` public class GibsonBruckSimulator extends Simulator ```

Implementation of the algorithm of Gibson and Bruck. They made two major improvements
to Gillespie's Direct method (`GillespieSimple`):

1. using a dependency graph
2. using a indexed priority queue

The first describes, which propensities have to be recalculates after some reaction
has fired, the latter puts an end to the time consuming search for the Direct
which fires after one firing. The priority queue just yields the next reaction to fire (from
which the name "Next reaction method" is derived). The priority queue holds for each
reaction the time of its next firing.

In order to do that the perspective of viewing the simulators time has to be changed
from relative to absolute. As a consequence, a new random number has to be drawn for
each reaction whose propensity has changed by a firing. To avoid this inefficient permanent
discarding of random numbers (drawing a random number is a time expensive operation)
Gibson and Bruck developed a method for reusing unused random numbers.

One pitfall remains: What happens, when a propensity decreases to 0 and after a time
ceases to be 0. Then the propensity from the pre-0 state can be reused which is tricky
to implement. Some implementations (including Dizzy) just draw a new random number which
is always valid but slow. Actually may these extra random numbers spoil the whole
improvement of the indexed priority queue in certain networks and it would be faster to
use the Direct method with the dependency graph (as in `GillespieEnhanced` ).

For reference see M.A.Gibson and J.Bruck, Efficient Exact Stochastic Simulation of Chemical
Systems with Many Species and Many Channels, J.Phys.Chem.A., Vol 104, no 9, 2000

**Author:**
:   Florian Erhard

**See Also:**: `GillespieSimple`, `GillespieEnhanced`, `DependencyGraph`, `IndexedPriorityQueue`

---

**Nested Class Summary**

**Nested classes/interfaces inherited from class fern.simulation.Simulator**

`Simulator.FireType`


**Field Summary**

**Fields inherited from class fern.simulation.Simulator**

`a, stochastics, t`


**Constructor Summary**

`GibsonBruckSimulator(Network net)`


| **Method Summary** | |
| --- | --- |
| `String` | `getName()`             Gets the name of the algorithm. |
| `void` | `initialize()`             Initializes the algorithm: set t=0reset the `AmountManager`recalculate the propensities Gets called at the very beginning of `start` |
| `void` | `performStep(SimulationController control)`             Performs one simulation step. |
| `void` | `reinitialize()`             Reset propensities when a event has been executed. |
| `void` | `setAmount(int species, long amount)`             Sets the amount of the given species. |
| `void` | `setVolume(double volume)`             Sets the volume of the reaction network. |

**Methods inherited from class fern.simulation.Simulator**

`addObserver, fireReaction, fireReaction, getAmount, getAmountManager, getNet, getNextThetaEvent, getPropensity, getPropensityCalculator, getTime, getVolume, isInterpolateTheta, registerNewTheta, setInterpolateTheta, start, start, thetaEvent`

**Methods inherited from class java.lang.Object**

`clone, equals, finalize, getClass, hashCode, notify, notifyAll, toString, wait, wait, wait`

**Constructor Detail**

### GibsonBruckSimulator

```
public GibsonBruckSimulator(Network net)
```


**Method Detail**

### reinitialize

```
public void reinitialize()
```

:   **Description copied from class: `Simulator`**
:   Reset propensities when a event has been executed.

    :   **Specified by:**: `reinitialize` in class `Simulator`

---


### performStep

```
public void performStep(SimulationController control)
```

:   **Description copied from class: `Simulator`**
:   Performs one simulation step. Between steps the terminating condition is checked.
    The simulators controller is passed, if an extending class wants to check it
    within one step. Implementing algorithms cannot be sure that the propensities
    are correct at the beginning of a step (e.g. if the volume changed). They should
    override `Simulator.setAmount(int, long)` and `Simulator.setVolume(double)`
    if they need correct values!

    :   **Specified by:**: `performStep` in class `Simulator`
    :   **Parameters:**: `control` - the simulators controller

---


### initialize

```
public void initialize()
```

:   **Description copied from class: `Simulator`**
:   Initializes the algorithm:

    - set t=0
    - reset the `AmountManager`
    - recalculate the propensities

    Gets called at the very beginning of `start`

    :   **Overrides:**: `initialize` in class `Simulator`

---


### getName

```
public String getName()
```

:   **Description copied from class: `Simulator`**
:   Gets the name of the algorithm.

    :   **Specified by:**: `getName` in class `Simulator`
    :   **Returns:**: name of the algorithm

---


### setVolume

```
public void setVolume(double volume)
```

:   **Description copied from class: `Simulator`**
:   Sets the volume of the reaction network.

    :   **Overrides:**: `setVolume` in class `Simulator`
    :   **Parameters:**: `volume` - the volume

---


### setAmount

```
public void setAmount(int species,
                      long amount)
```

:   **Description copied from class: `Simulator`**
:   Sets the amount of the given species. Propensities have to be recalculated!

    :   **Overrides:**: `setAmount` in class `Simulator`
    :   **Parameters:**: `species` - species index: `amount` - amount of species **See Also:**: `AmountManager.setAmount(int, long)`


---


|  |  |  |
| --- | --- | --- |
| **Overview** \| **Package** \| **Class** \| **Use** \| **Tree** \| **Deprecated** \| **Index** \| **Help** | |  |
| **PREV CLASS**   **NEXT CLASS** | **FRAMES**    **NO FRAMES**     **All Classes** |  |
| SUMMARY: NESTED \| FIELD \| CONSTR \| METHOD | DETAIL: FIELD \| CONSTR \| METHOD |  |


---
